# Maternal Anxiety and Psychosocial Burden in Childhood Atopic Dermatitis: A Comparative Study

**DOI:** 10.62641/aep.v54i1.2048

**Published:** 2026-02-15

**Authors:** Elif Küçük, Fatih Çiçek

**Affiliations:** ^1^Department of Psychiatry, Yüksek Ihtisas University, 06530 Ankara, Turkey; ^2^Department of Pediatrics, Istinye University, 34396 Istanbul, Turkey

**Keywords:** atopic dermatitis, quality of life, anxiety, caregiver burden, mothers, SCORAD

## Abstract

**Background:**

Atopic dermatitis (AD) is among the most prevalent chronic pediatric skin diseases. Beyond its cutaneous manifestations, AD imposes substantial psychosocial and economic strain on families. We quantified this burden—i.e., maternal anxiety and quality of life (QoL)—and identified its clinical and sociodemographic determinants, comparing families of children with AD to those of healthy peers.

**Methods:**

This cross-sectional study enrolled 84 mothers of physician-diagnosed patients with AD aged 3–144 months and 90 mothers of age-matched healthy children attending routine visits. Disease severity was graded with the Scoring Atopic Dermatitis (SCORAD) index. Mothers completed the State-Trait Anxiety Inventory (STAI) and the 8-item European Health Impact Scale (EUROHIS-QoL). Additionally, the Dermatological Family Impact Scale (DeFIS) was administered to the AD group.

**Results:**

EUROHIS-QoL scores were lower in the AD group than in controls (*p* = 0.016), whereas The State-Anxiety Inventory (STAI-S) scores were higher (*p* < 0.001); the Trait-Anxiety Inventory (STAI-T) scores did not differ (*p* = 0.125). In multivariable models, patient status (*p* = 0.006), higher STAI-T (*p* = 0.002), and greater income (USD 750–1500: *p* = 0.014; >USD 1500: *p* = 0.010) were independently associated with QoL. Anxiety was driven by patient status, lower QoL, and higher STAI-T, while trait anxiety was driven by lower QoL and higher STAI-S. SCORAD correlated negatively with QoL (ρ = –0.225; *p* = 0.040) and positively with STAI-S and DeFIS.

**Conclusions:**

Pediatric AD significantly impairs mothers' QoL and heightens maternal situational anxiety and these effects intensify with increasing disease severity and financial strain. Multidisciplinary, family-centered care, including psychological screening and targeted support for low-income households, is essential for comprehensive AD management.

## Introduction

Atopic dermatitis (AD) is among the most common chronic skin diseases of childhood, posing a considerable burden on affected individuals and their families. AD is a relapsing inflammatory skin disorder with a pathogenesis that involves environmental, genetic, and immunological factors, and characterized by erythema, pruritus, xerosis, and eczematous plaques [1, 2]. The prevalence of AD is 2.5%–30.0% in children, and has been increasing in industrialized countries in recent years [3, 4, 5]. Symptoms typically begin in early childhood, with 60% of cases presenting before age 1 year and the majority before age 5 years [6, 7].

In children, AD causes pruritus, pain, and diminished sleep quality, leading to a range of social consequences, such as reduced school performance and low self-esteem [8]. In a study of pediatric chronic diseases, Beattie and Lewis-Jones [9] showed that, after cerebral palsy, AD is the condition that most severely impairs quality of life (QoL). Families of affected children likewise have sleep disturbances, stress, anxiety, depression, impaired daytime functioning, and poorer QoL owing to economic pressures [10, 11, 12].

Recent studies have increasingly emphasized the disproportionate psychological burden experienced by mothers of children with AD. Compared with mothers of healthy children, these caregivers report significantly higher levels of state anxiety, depressive symptoms, and emotional exhaustion [12, 13]. In a Turkish study, Kilic and Kilic [12] found that anxiety scores measured by the Beck Anxiety Inventory were significantly elevated in mothers of children with AD. This increase in maternal anxiety was accompanied by higher caregiver burden and reduced QoL. Furthermore, existing literature suggests that the severity of the child’s disease—rather than the child’s age or duration of illness—is the most crucial determinant of maternal psychological distress. In particular, uncontrolled disease flares are associated with marked increases in maternal anxiety levels [13]. In a study conducted by Pustišek *et al*. [14], mothers of children with AD were identified as the primary caregivers and were shown to bear the greatest share of emotional and psychosocial burden within the family. Taken together, these findings underscore the importance of considering maternal mental health not merely as a secondary outcome, but as a clinically relevant and independent variable in pediatric AD research.

Several investigations have confirmed that the financial burden of prolonged treatment and care further amplifies emotional stress within families and diminishes overall QoL [15, 16]. In an international study spanning 18 countries, Barbarot *et al*. [17] underscored that greater AD severity intensifies the physical, emotional, social, and economic burden on families. Moreover, supportive education and behavioral modifications have been shown to lessen disease severity in children, thereby alleviating the psychological burden on both the child and the parents [18]. Despite growing evidence, the joint impact of disease severity and socioeconomic factors on maternal anxiety and family QoL remains underexplored, especially in middle-income settings.

Accordingly, this study aims to (i) quantify the psychological burden (i.e., maternal anxiety and QoL) in caregivers of children with AD and identify associated clinical and socio-demographic factors, and (ii) compare these outcomes with those of mothers of healthy children.

## Methods

### Study Design and Participants

We conducted this cross-sectional study between September 1, 2023 and May 1, 2024 at the Paediatric Allergy and Immunology Clinic, Kartal Dr Lütfi Kırdar City Hospital. Eligible participants were mothers of children aged 3–144 months who had been diagnosed with AD by a pediatric allergy and immunology specialist according to the Hanifin–Rajka criteria, and mothers of age-matched children presenting to the General Pediatrics Clinic for routine care (e.g., vaccination and growth monitoring) without acute complaints [19]. Only mothers who were literate and fluent in Turkish were included in the study.

Exclusion criteria for the study were the presence of metabolic disorders, immunodeficiency, chronic heart disease, endocrine, renal, pulmonary, gastrointestinal, hepatic, or central nervous system diseases, malignancies, or any other chronic conditions in the child or other family members. In addition, mothers with an active psychiatric disorder or those using psychiatric medications, and mothers who declined to participate in the study were excluded. Since local corticosteroid treatment can influence AD severity and Scoring Atopic Dermatitis (SCORAD) index scores, patients who had received such treatment within the past 4 weeks were also excluded. The study commenced after eligible participants were informed about the study and written informed consent was obtained.

A priori power analysis was performed using G*Power software (version 3.1.9.7, Düsseldorf, Germany). Assuming a medium effect size (d = 0.50), an alpha level of 0.05, and a power of 0.90, the required sample size was 172 participants (86 per group). To account for an anticipated 10% dropout rate, nine additional participants were planned for each group. During data collection, 11 participants from the patient group and five from the control group were lost at follow-up. The final analytic sample consisted of 84 patients and 90 control patients.

Parents first completed a questionnaire for demographic information, followed by the State-Trait Anxiety Inventory (STAI) and the European Health Impact Scale (EUROHIS-QoL). In addition, mothers of children with AD also completed the Dermatological Family Impact Scale (DeFIS). The study was approved by the Ethics Committee of Kartal Dr. Lutfi Kirdar City Hospital (decision number: 2023/514/250/28, date May 29, 2023) and was conducted in accordance with the principles of the Declaration of Helsinki.

### SCORAD Index

Disease severity was evaluated using the SCORAD index, a standardized instrument developed by the European Task Force on AD to quantify the severity of AD. The index integrates both objective and subjective components, including the extent of skin involvement, the intensity of six clinical signs (i.e., erythema, edema/papulation, oozing/crusting, excoriation, lichenification, and dryness), and two patient-reported symptoms—pruritus and sleep disturbance—each scored on a 0–10 visual analogue scale. The total score ranges from 0 to 103, with higher scores indicating greater disease severity [20]. Based on the recent interpretation proposed by Barbarot *et al*. [21], disease severity was categorized as clear/almost clear (<12), mild (12–25), or moderate/severe (≥25) according to the SCORAD index. This index has been validated for both clinical and research use and shows a high interobserver reliability [20, 21].

### Spielberger STAI

The scale developed by Spielberger *et al*. [22] was validated in Turkish by Öner [23]. This scale consists of two subscales, each containing 20 items designed to measure state and trait anxiety. The State-Anxiety Inventory (STAI-S) assesses how an individual feels at a specific moment and under certain conditions, while the Trait-Anxiety Inventory (STAI-T) evaluates how an individual feels. Scores on the scale range from 20 to 80, with higher scores on each subscale indicating a greater anxiety. The Cronbach’s α internal consistency coefficients of the inventory were 0.889 for the STAI-S and 0.732 for the STAI-T subscales, respectively.

### EUROHIS-QoL

The EUROHIS-QoL is an 8-item instrument derived from the World Health Organization Quality of Life (WHOQoL) scale. The scale evaluates multiple domains of wellbeing, including overall QoL, perceived health, energy levels, autonomy in daily functioning, self-esteem, interpersonal relationships, financial status, and living conditions. Each item is rated on a scale from 0 (not at all) to 5 (completely) [24]. The Turkish adaptation and psychometric validation of the scale were conducted by Eser *et al*. [25] in 2010. The total score on the scale ranges from 8 to 40, with higher scores indicating better QoL. The Cronbach’s α coefficient for the scale was 0.811.

### The DeFIS

The DeFIS that was designed by Turan *et al*. [26] for the Turkish population, is a 15-item scale that evaluates the past month. Each item is scored from 0 to 4, with five response options ranging from “always” to “never”. The scale was validated for reliability and is deemed appropriate for assessing the psychological, social, physical, financial, and daily life impacts of various chronic dermatoses on family members [26]. The Cronbach’s α coefficient for the scale was 0.853 for the overall scale.

### Statistical Analysis

Data were analyzed using IBM SPSS Statistics, version 30 (IBM Corp., Armonk, NY, USA). Descriptive statistics were expressed as frequency (n), percentage (%), mean ± standard deviation, median, and minimum–maximum values, as appropriate to the data type. The normality of continuous variables were assessed using the Shapiro–Wilk test. For between-group comparisons, the Mann–Whitney U test was applied and the Kruskal–Wallis test for comparisons involving more than two groups. Bonferroni adjustment was applied to all pairwise post-hoc analyses following a significant Kruskal–Wallis result. Associations between continuous variables were evaluated using Spearman’s rank correlation coefficient. For categorical variables, intergroup comparisons were made using Chi-squared tests (Pearson’s Chi-squared, Fisher–Freeman–Halton exact, or Yates-corrected Chi-squared, as appropriate). When a Chi-squared test yielded statistical significance, post-hoc subgroup analyses were conducted using Bonferroni-corrected two-proportion Z tests. To identify independent predictors of scale scores, multiple linear regression analyses were performed. Variables with *p* < 0.25 in univariate analyses were included in the multivariate model; categorical predictors were coded as dummy variables. The final model was determined by backward elimination. Multicollinearity was evaluated using variance inflation factors, and 95% confidence intervals were calculated for all regression coefficients. A two-tailed *p* < 0.05 was considered statistically significant.

## Results

84 children were included in the patient group and 90 in the control group. Girls made up 52.4% of the patient group and 45.6% of the controls, with no significant difference in sex distribution (*p* > 0.05). The groups showed statistically similar distributions with respect to child age, maternal age, and number of children (*p* > 0.05). The proportion of mothers with a university degree was 71.4% in the patient group and 67.8% in the control group, again showing no significant difference. Employment status was also similar, with 28.6% of mothers in the patient group and 18.9% in the control group being employed. STAI-S scores were significantly higher among mothers in the patient group than those in the control group (*p* < 0.001), whereas STAI-T scores did not differ (*p* = 0.125). EUROHIS-QoL scores were significantly higher in mothers from the control group than in those from the patient group (*p* = 0.016; Table 1).

**Table 1.** **Comparison of sociodemographic characteristics of the patient and control groups**.

Variable		Groups		Test statistic	
		Patient (n = 84)	Control (n = 90)	Test value	*p* value
Sex, n (%)				0.810	0.368^‡^
	Female	44 (52.4%)	41 (45.6%)		
	Male	40 (47.6%)	49 (54.4%)		
Child age, months		30.5 (3–140)	33.5 (4–126)	0.288	0.774^&^
Mother’s age, years		30.5 (21–44)	31 (20–49)	0.130	0.897^&^
Number of children		2 (1–4)	2 (1–5)	1.538	0.124^&^
Maternal education, n (%)				5.250	0.132^¥^
	Primary school	0 (0%)	3 (3.3%)		
	Middle school	11 (13.1%)	18 (20.0%)		
	High school	13 (15.5%)	8 (8.9%)		
	University	60 (71.4%)	61 (67.8%)		
Maternal employment, n (%)				1.756	0.185^Φ^
	Employed	24 (28.6%)	6 (18.9%)		
	Unemployed	60 (71.4%)	73 (81.1%)		
Monthly household income, n (%)				34.446	< **0.001^‡^**
	<USD 750	13 (15.5%)	27 (30.0%)		
	USD 750–1500	49 (58.3%)	14 (15.6%)		
	>USD 1500	22 (26.2%)	49 (54.4%)		
State-anxiety score		45 (20–63)	37 (21–55)	4.212	< **0.001^&^**
Trait-anxiety score		43 (28–59)	42 (24–61)	1.533	0.125^&^
Quality-of-life score		26 (12–35)	28.5 (12–37)	2.414	**0.016^&^**

n, number of patients; %, column percentage. Continuous variables are expressed as median (minimum–maximum). ^‡^ Pearson’s Chi-squared test. ^¥^ Fisher–Freeman–Halton exact test. ^Φ^ Yates-corrected Chi-squared test. ^&^ Mann–Whitney U test. Statistically significant (*p* < 0.05) values in bold.

Of the children in the patient group, the most frequently reported manifestations were erythema (100%) and pruritus (96.4%). Restlessness (57.1%) and rash (48.8%) were also common, whereas gastrointestinal complaints, including vomiting (3.6%), constipation (6.0%), diarrhea (9.5%), and abdominal pain (6.0%), were noted in a smaller proportion of patients. Severe respiratory events, such as dyspnea (2.4%) and anaphylaxis (2.4%) were infrequent. The median SCORAD score was 26.5 (range 5.3–53.0), consistent with an overall moderate level of disease severity. Based on the SCORAD categories, 57.1% of patients were classified as moderate/severe, 34.5% as mild, and 8.3% as clear/almost clear. Total DeFIS scores ranged from 3 to 59, with a median of 27 (Table 2). As these variables were assessed only in the patient group, they were not included in the regression or between-group analyses.

**Table 2.** **Clinical characteristics of children with atopic dermatitis**.

Variables		Value, n (%)
Erythema		84 (100%)
Pruritus		81 (96.4%)
Restlessness		48 (57.1%)
Rash		41 (48.8%)
Vomiting		3 (3.6%)
Constipation		5 (6.0%)
Diarrhea		8 (9.5%)
Abdominal pain		5 (6.0%)
Dyspnea		2 (2.4%)
Anaphylaxis		2 (2.4%)
Disease duration, months		18 (2–120)
SCORAD total		26.5 (5.3–53.0)
SCORAD category		
	Clear/almost clear	7 (8.3%)
	Mild	29 (34.5%)
	Moderate/severe	48 (57.1%)
DeFIS total		27 (3–59)

DeFIS, Dermatological Family Impact Scale; SCORAD, Scoring Atopic Dermatitis. Median (minimum–maximum) for continuous variables; n, number of patients.

Table 3 presents the distribution of scale scores across study variables. EUROHIS-QoL scores were higher in the control group than in the patient group (*p* = 0.016). STAI-S scores were significantly elevated in the patient group (*p* < 0.001), whereas STAI-T scores did not differ between groups (*p* = 0.125). EUROHIS-QoL scores correlated negatively with STAI-S (ρ = –0.311, *p* < 0.001) and negatively with STAI-T (ρ = –0.295, *p* < 0.001). STAI-S and STAI-T were themselves moderately and positively correlated (ρ = 0.402, *p* < 0.001). No significant associations were found between disease duration and any psychosocial scale (all *p* > 0.30). Mothers with a monthly household income >USD 1500 showed higher EUROHIS-QoL scores and lower anxiety scores than those in the <USD 750 and USD 750–1500 categories (QoL H = 5.900, *p* = 0.043; STAI-S H = 6.901, *p* = 0.032; STAI-T H = 6.654, *p* = 0.036). No other variables were significantly associated with the scale scores.

**Table 3.** **Comparison of scale scores by variables**.

		EUROHIS-QoL	STAI-S	STAI-T
Group				
	Patient	26 (24–29)	45 (35.75–50)	43 (38–48)
	Control	28.5 (24–31)	37 (31–44.75)	42 (37–45)
	*z*; *p* value	2.414; **0.016**	4.212; <**0.001**	1.533; 0.125
Sex				
	Female	26 (24–30)	41 (29–47)	42 (38–46)
	Male	27 (23–30)	41 (34–47)	42 (36–47)
	*z*; *p* value	0.551; 0.581	0.985; 0.324	0.591; 0.555
Child age (*rho*; *p* value)		0.055; 0.474	–0.062; 0.414	–0.005; 0.948
Maternal age (*rho*; *p* value)		–0.117; 0.124	–0.019; 0.806	0.142; 0.061
Number of children (*rho*; *p* value)		–0.115; 0.132	–0.094; 0.220	0.034; 0.658
Maternal education				
	Primary school	22 (17–24)	40 (37.5–42)	49 (43.5–55)
	Middle school	28 (24–31)	41 (34–42)	43 (39–44)
	High school	26 (21–29)	45 (32–50)	40 (38–46)
	University	27 (24–30)	43 (33–47)	42 (37–47)
	*H*; *p* value	4.828; 0.185	1.667; 0.644	1.993; 0.574
Maternal employment				
	Yes	27 (24–29)	42 (37–47)	45 (39–47)
	No	27 (24–30)	41 (32–47)	42 (37–46)
	*z*; *p* value	0.023; 0.982	0.715; 0.475	1.360; 0.174
Monthly household income				
	<USD 750	25 (23.5–29) ^a^	40 (31.8–44) ^a^	42 (39–46.3) ^a⁢b^
	USD 750–1500	26 (24–29) ^a^	45 (35–50) ^b^	44 (38–48.5) ^a^
	>USD 1500	28 (24–31) ^𝑏^	41 (32.5–46) ^a⁢b^	40 (36–45) ^b^
	*H*; *p* value	5.90; **0.043**	6.901; **0.032**	6.654; **0.036**
Disease duration (*rho*; *p* value)		0.008; 0.945	–0.111; 0.317	0.064; 0.561
EUROHIS-QoL (*rho*; *p* value)		-	–0.311; <**0.001**	–0.295; <**0.001**
STAI-S (*rho*; *p* value)		–0.311; <**0.001**	-	0.402; <**0.001**
STAI-T (*rho*; *p* value)		–0.295; <**0.001**	0.402; <**0.001**	-

n, number of patients; %, column percentage, continuous variables are presented as median (25th–75th percentile); z, Mann–Whitney U test; H, Kruskal–Wallis H test; ρ, Spearman’s correlation coefficient; EUROHIS-QoL, European Health Impact Scale; STAI-S, state-anxiety inventory; STAI-T, trait-anxiety inventory. Superscripts a and b within the same column denote categories that differ significantly, categories sharing the same superscript do not differ significantly. Statistically significant (*p* < 0.05) values in bold.

To identify the final set of factors influencing each scale score, variables with *p* < 0.25 in the univariate analyses (Table 3) were entered into separate multiple linear regression models for each outcome. Categorical variables were incorporated using dummy coding, and the final models were derived with backward elimination. The regression results are presented in Table 4.

**Table 4.** **Assessment of factors influencing scale scores using multiple linear regression analysis**.

		Regression coefficients							VIF
		β	SE	Std. β	t	*p*	95% confidence interval for β		
							Lower	Upper	
**Model 1: factors associated with quality-of-life scores**									
Intercept		34.325	2.561		13.405	<0.001	29.270	39.380	
Group									
	Control	Reference							
	Patient	–2.247	0.814	–0.222	–2.760	0.006	–3.855	–0.640	1.250
STAI-T		–0.168	0.053	–0.230	–3.148	0.002	–0.273	–0.062	1.049
Monthly household income									
	<USD 750	Reference							
	USD 750–1500	2.541	1.026	0.241	2.477	0.014	0.516	4.566	1.880
	>USD 1500	2.458	0.946	0.239	2.597	0.010	0.590	4.326	1.673
Variables entered into the model: group, state-anxiety scores, trait-anxiety scores, maternal age, number of children, educational level and, income level. Model statistics: F = 6.019; *p * < 0.001; R^2^ = 0.152; adjusted R^2^ = 0.127.									
**Model 2: factors associated with state-anxiety scores**									
Intercept		29.172	6.053		4.820	<0.001	17.224	41.120	
Group									
	Control	Reference							
	Patient	4.891	1.315	0.251	3.720	<0.001	2.295	7.487	1.039
EUROHIS-QoL		–0.362	0.133	–0.188	–2.722	0.007	–0.625	–0.100	1.072
STAI-T		0.433	0.096	0.308	4.505	<0.001	0.244	0.623	1.092
Variables entered into the model: group, quality-of-life scores, trait-anxiety scores, number of children, and income level. Model statistics: F = 19.629; *p * < 0.001; R^2^ = 0.257; adjusted R^2^ = 0.244.									
**Model 3: factors associated with trait-anxiety scores**									
Intercept		38.635	3.790		10.195	<0.001	31.155	46.116	
EUROHIS-QoL		–0.256	0.098	–0.188	–2.624	0.009	–0.449	–0.064	1.106
STAI-S		0.251	0.051	0.356	4.956	<0.001	0.151	0.352	1.106
Variables entered into the model: group, quality-of-life scores, state-anxiety scores, maternal age, employment status, and income level. Model statistics: F = 21.842; *p * < 0.001; R^2^ = 0.203; adjusted R^2^ = 0.194.									

EUROHIS-QoL, European Health Impact Scale; STAI-S, state-anxiety inventory; STAI-T, trait-anxiety inventory. Bold text indicates results from different regression models.

According to Table 4, group status, STAI-T score, and income level were the variables that remained major predictors of EUROHIS-QoL scores. Membership in the patient group was associated with a significant reduction in EUROHIS-QoL (β = –2.247; *p* = 0.006). Higher STAI-T scores were also linked to lower EUROHIS-QoL (β = –0.168; *p* = 0.002). With respect to income, EUROHIS-QoL scores were significantly higher both in the USD 750–1500 (β = 2.541; *p* = 0.014) and >USD 1500 categories (β = 2.458; *p* = 0.010) relative to the <USD 750 reference category. The model explained a modest proportion of the variance (adjusted R^2^ = 0.127) and was significant overall (F = 6.019; *p* < 0.001).

Model 2 examined the determinants of STAI-S scores. Membership in the patient group was associated with significantly higher STAI-S scores (β = 4.891; *p* < 0.001). In contrast, higher EUROHIS-QoL scores were associated with lower STAI-S scores (β = –0.362; *p* = 0.007), whereas higher STAI-T scores were associated with higher STAI-S scores (β = 0.433; *p* < 0.001). The model accounted for a substantial proportion of the variance (adjusted R^2^ = 0.244) and was significant overall (F = 19.629; *p* < 0.001).

Model 3 investigated the factors associated with STAI-T scores. Higher EUROHIS-QoL scores were linked to a significant reduction in STAI-T (β = –0.256; *p* = 0.009), whereas higher STAI-S scores exerted a positive—i.e., aggravating—effect on STAI-T (β = 0.251; *p* < 0.001). The model was significant overall (F = 21.842; *p* < 0.001) and accounted for a moderate proportion of the variance (adjusted R^2^ = 0.194). Collectively, these findings indicate an inverse relationship between QoL and anxiety, and show that being in the patient group both diminishes QoL and elevates anxiety. Moreover, rising income was linked to better QoL, suggesting that socioeconomic conditions play an important role in psychological wellbeing.

Table 5 summarizes correlation analyses conducted solely within the AD group to evaluate the relationship between clinical severity and psychosocial outcomes. Specifically, SCORAD scores were significantly and inversely correlated with EUROHIS-QoL scores (ρ = –0.225; *p* = 0.040), indicating that greater AD severity was accompanied by poorer EUROHIS-QoL. SCORAD scores were also positively and significantly correlated with STAI-S levels (ρ = 0.218; *p* = 0.048), suggesting that higher disease severity might be linked to heightened situational anxiety. No significant relationship was observed between SCORAD and STAI-T scores (ρ = 0.059; *p* = 0.595), implying that disease severity was more closely related to a momentary affect than to long-term anxiety disposition. Lastly, SCORAD scores correlated positively and significantly with DeFIS scores (ρ = 0.354; *p* = 0.001), demonstrating that as AD severity increases, the degree of family burden rises accordingly.

**Table 5.** **Association between SCORAD scores and other scale scores in the patient group**.

	SCORAD
	*rho*; *p* value
EUROHIS-QoL	–0.225; **0.040**
STAI-S	0.218; **0.048**
STAI-T	0.059; 0.595
DeFIS	0.354; **0.001**

ρ, Spearman’s correlation coefficient; DeFIS, Dermatology Family Impact Scale; EUROHIS-QoL, European Health Impact Scale; SCORAD, Scoring Atopic Dermatitis; STAI-S, state-anxiety inventory; STAI-T, trait-anxiety inventory. Statistically significant (*p* < 0.05) values in bold.

## Discussion

Our study shows that childhood AD is associated with reduced family QoL and elevated maternal situational anxiety. Previous research has similarly shown that families of children with AD experience substantial psychosocial burden and notable reductions in QoL, with these effects particularly pronounced among mothers [27, 28, 29]. Chronic manifestations of AD—particularly pruritus and sleep disruption—are linked to poorer academic performance and reduced self-esteem in affected children, while simultaneously fragmenting parental sleep and precipitating fatigue, anxiety, and caregiver burnout [8, 12]. In a qualitative study, Capozza *et al*. [10] found that mothers of children with AD reported persistent exhaustion and overwhelm, accompanied by a perceived inadequacy in their maternal role. Collectively, these findings underscore that AD is not merely a dermatological condition, rather, it profoundly affects the daily lives of affected families with mothers bearing a considerable share of the psychosocial impact.

In our cohort, mothers of children with AD reported significantly lower QoL scores than mothers of healthy children, underscoring that AD affects both the patient and the entire family. A Turkish study by Kilic and Kilic [12] also showed that both children with AD and their mothers experienced diminished QoL attributable to the disease. Moreover, another study reported that mothers of children with AD frequently had difficulty falling asleep, had perceived poor sleep quality, and had persistent daytime fatigue [30]. A growing body of research has further established that greater disease severity—as quantified by higher SCORAD scores—is associated with more pronounced impairments in family QoL [31, 32]. Consistent with these findings, we observed a negative correlation between SCORAD scores and overall family QoL; as AD severity increased, family QoL decreased. Further to this trend, family impact scores on the DeFIS increased in parallel with AD severity. In an international study of 7645 child–parent/caregiver dyads across 18 countries, Barbarot *et al*. [17] found that escalating disease severity substantially amplified the physical, emotional, social, and economic burden on families. Similar results were reported by Siafaka *et al*. [33], who emphasized that the influence of AD on both child and family QoL is closely linked to SCORAD-defined disease severity. Collectively, these data support a proportional degradation in family QoL as AD severity worsens.

The psychological impact on mothers of children with AD was also pronounced, and our findings are consistent with the existing literature. Mothers in the patient group exhibited significantly higher STAI-S scores than those in the control group, whereas STAI-T scores did not differ between groups. This pattern suggests that maternal anxiety in the context of childhood AD is driven more by situational stress related to the disease than by a stable dispositional tendency. Previous reports also indicate that mothers of children with AD have higher levels of anxiety and stress than mothers of healthy children [34, 35]. Supporting these findings, Song *et al*. [34] evaluated 120 parent–child dyads across mild, moderate, and severe AD subgroups versus healthy controls using standardized measures of anxiety and depression. They reported significantly higher anxiety scores among mothers of children with AD compared with controls, while mothers in the moderate and severe AD groups also showed significantly elevated depression scores. Likewise, a large-scale South Korean study including 970 children with AD and 5733 without AD found that mothers of children with AD had higher levels of perceived stress and an increased tendency toward suicidal ideation compared with mothers of children without AD [35]. In our dataset, disease severity was positively associated with maternal STAI-S, indicating a modest but significant association between higher disease severity and increased maternal situational distress; however, this relationship should be interpreted cautiously due to the small effect size. By contrast, STAI-T remained unaffected, implying that baseline anxiety propensity is similar between groups, but flares in the child’s disease promote sharp increases in situational anxiety among mothers. Thus, AD appears to function as a salient environmental stressor that might be associated with transient increases in maternal stress responses.

In our study, neither disease duration nor patient age showed a significant association with QoL or anxiety scores, a finding that is consistent with the literature [36, 37]. Previous reports emphasize that disease severity—rather than age or sex—is the primary factor that adversely affects QoL [17, 37]. Thus, the evidence indicates that severity is crucial, independent of how long the disease has been present or the child’s age. However, the influence of disease duration has been inconsistently reported. A study published in 2023 found significantly higher maternal anxiety and depression when children had lived with AD for more than 6 months, suggesting that chronicity might impose a cumulative psychological burden on caregivers [27]. By contrast, Su *et al*. [13] observed that among Chinese mothers, anxiety risk declined slightly as the duration of their child’s AD increased—possibly reflecting the development of coping mechanisms—while severe disease remained strongly associated with heightened anxiety. These divergent findings imply a complex balance between adaptation to a chronic illness and accumulated caregiver burden. Lastly, the limited influence of child sex on QoL and anxiety in our cohort corroborates previous work showing no significant sex-related differences in QoL among children with AD [38, 39].

Our study revealed significant differences in anxiety scores across income strata, with anxiety levels being highest among mothers in the lowest income group. This finding suggests that economic hardship functions as an additional stressor in coping with a chronic condition, such as AD. Families of children with AD face substantial financial pressures stemming from ongoing treatment expenses, frequent clinic visits, loss of work productivity, and the out-of-pocket cost of moisturizers and dermatological cosmetic products that are not reimbursed by national insurance. Filanovsky *et al*. [15] showed that childhood AD imposes a pronounced financial and emotional burden on families. Similarly, a large multicenter study in Europe underscored that moderate-to-severe AD generates considerable direct costs and productivity losses not only for patients, but also for their families and society at large [16]. Our findings align with these studies, indicating that families with low-income are disproportionately affected by the care-related expenses and lifestyle adjustments required by AD. Accordingly, economic assistance programs and cost-effective treatment strategies are crucial for safeguarding psychosocial wellbeing in this at-risk population.

Our findings carry several implications for both everyday practice and community health. First, the management of AD should address both cutaneous manifestations and the disorder’s psychosocial impact on the family. Optimizing the child’s dermatological treatment should improve QoL for the entire household. Indeed, Barbarot *et al*. [17] showed that reducing disease severity alleviates the physical and emotional burden borne by families. In this context, clinicians should routinely screen parents of children with AD for psychological distress and, where appropriate, refer them for support. As underscored by Kobusiewicz *et al*. [27], assessing the degree of functional impairment in mothers—and providing targeted assistance—is crucial for both maternal and child health. Parents with anxiety or depressive symptoms could struggle to adhere to the child’s treatment plan; studies have shown that caregiver depression is associated with significantly poorer adherence in pediatric patients [13]. Parental education programs have also been shown to improve AD management [18]. Such interventions can help caregivers gain better control over their child’s symptoms while simultaneously enhancing their own QoL. Consequently, routine evaluation—and, when necessary, treatment—of parental mental health might indirectly improve clinical outcomes in children with AD.

Moreover, emerging evidence suggests that maternal psychological distress during pregnancy could contribute to the risk and severity of allergic conditions in offspring. Specifically, antenatal anxiety and depression are implicated in the development and clinical expression of pediatric AD [40, 41]. These observations underscore the need to adopt a bidirectional framework in future research and highlight the importance of longitudinal studies to elucidate the causal direction and underlying mechanisms of this association.

This study had various methodological limitations. First, its cross-sectional design permitted only contemporaneous associations, limiting causal inference. Second, the single-center, hospital-based sampling strategy might have introduced selection bias and restricted the generalizability of the findings to the broader population. Third, by focusing exclusively on mothers, we were unable to evaluate the psychosocial burden of fathers or other family members. The absence of a treatment group limited the exploration of causal pathways between AD severity and maternal outcomes. Lastly, the wide age range of included children, though reflective of clinical reality, might have introduced developmental variability affecting maternal responses.

Our investigation also had notable strengths. Disease severity was assessed objectively with the SCORAD index; socioeconomic factors (e.g., income) were statistically controlled with multivariable regression; and all of the instruments used have validated Turkish versions. Moreover, the study adds valuable data from Türkiye, where evidence remains scarce. A further unique contribution is the separate assessment of state anxiety and trait anxiety in mothers, enabling a clearer delineation of the acute stress effect of childhood AD.

## Conclusions

This study shows that childhood AD markedly diminishes maternal QoL and, in particular, elevates maternal state anxiety. Our findings indicate that greater disease severity is associated with higher family burden, whereas disease duration does not appear to shape psychosocial outcomes. The association between low household income and heightened anxiety further confirms that financial constraints constitute an additional stressor. Considering these findings, the implementation of targeted interventions (e.g., structured psychoeducational programs [42], cognitive–behavioral approaches [43], and digital mental health tools [44]) might offer substantial benefits for mothers with low income or elevated anxiety levels. Moreover, community-based support initiatives could serve as cost-effective strategies to reduce psychosocial burden and enhance adherence to pediatric AD treatment protocols [45]. Such multifaceted interventions reportedly improve both caregiver wellbeing and disease management outcomes [42, 43, 44, 45]. Future longitudinal studies are warranted to explore causal pathways and to incorporate fathers and other caregivers for a more comprehensive understanding of family-wide psychosocial impact.

## Availability of Data and Materials

The datasets generated and analyzed during the current study are not publicly available due to privacy and ethical restrictions but are available from the corresponding author on reasonable request.
